# Multivalent cooperativity induced by self-assembly for f-element separation

**DOI:** 10.1038/s42004-021-00514-1

**Published:** 2021-05-28

**Authors:** Lixi Chen, Shuao Wang

**Affiliations:** State Key Laboratory of Radiation Medicine and Protection, School for Radiological and Interdisciplinary Sciences, and Collaborative Innovation Center of Radiation Medicine of Jiangsu Higher Education Institutions, Soochow University, Suzhou, China

**Keywords:** Nuclear chemistry, Coordination chemistry, Self-assembly

## Abstract

Preorganization is an effective strategy for f-element separation, but the complexity of extractant synthesis hinders large-scale application. Here the authors discuss an alternative strategy induced by in situ self-assembly that borrows principles of multivalent cooperativity from Nature to separate f-elements.

## Background

The f-elements (lanthanides and actinides) are essential to modern society and have been widely used in magnets, luminescent materials, catalysis, the nuclear industry, and so on^1,2^. Nevertheless, the separation of f-elements represents a great challenge due to their chemical similarities^3^. For example, the lanthanide contraction leads to only a 0.15-Å decrease in ionic radii across the series. Such subtle differences make it a difficult task to separate neighboring lanthanides. Traditional liquid–liquid extraction approaches, which apply small organic extractants to form complexes with target metal ions, usually suffer from low separation efficiency, especially for elements with almost identical chemical and physical properties (e.g., lanthanides and minor actinides)^2,4^. A strategy termed as “preorganization,” where multiple ligating groups are integrated into a relatively rigid platform, can improve separation performance^4,5^.

Preorganization involves constructing a multidentate ligand that offers multiple donors with proper orientation for coordination^4–6^. The effect of preorganization resembles the multivalency and cooperativity seen in biological processes. Multivalency refers to interactions formed from several individual noncovalent bonds^7,8^ and tends to be dramatically stronger than the sum of the corresponding monovalent ones, as usually witnessed in the multi-binding adhesion of viruses to host cells^9^. Cooperativity describes how the binding of one ligand can influence subsequent binding equilibria in a stepwise binding event^7,10^. In this case, each module of an entity should be well assembled so as to work in a synergistic manner. A paradigmatic example in biology is the allosteric oxygenation of hemoglobin, which contains four hemes that serve as binding sites for oxygen. The binding of one oxygen can raise the affinity of the others^11^. Bearing the concept of multivalency and cooperativity in mind, one can rationally design preorganized extractants possessing multiple coordination sites with defined topology that match specific metal ion over undesired ones.

**Fig. 1:**
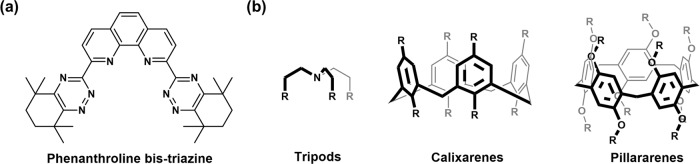
Chemical structures of some classical preorganization platforms for f-element separation. **a** Phenanthroline-derived bis-triazine^12^ and **b** tripods, calixarenes, and pillararenes^2,14,15^.

The current preorganization platforms can be roughly classified into two types: those with rigid planar skeletons and those with three-dimensional (3D) scaffold. The best-known example of the former class is the phenanthroline-derived bis-triazine ligand (Fig. 1a), where four N donors are precisely embedded into the rigid skeleton to coordinate Am^3+^ effectively^12,13^. In recent years, increasing efforts have been directed toward the design of preorganized ligands based on 3D scaffolds because they can fulfill the 3D coordination preference of f-elements. Tripods, calixarenes, resorcinarenes, and pillararenes are typical 3D scaffolds (Fig. 1b) for f-element separation^2,14,15^. However, the more the ligating groups are anchored, the more complicated the ligands are to synthesize. This greatly limits the large-scale production of extractants for industrial application, promoting the search for alternative methods for f-block separation.

## Recent advances

Recently, a strategy based on self-assembled systems was proposed for lanthanide or actinide separation^16–18^. Distinct from the preorganization strategy, where multiple ligating groups are covalently incorporated into a molecular platform, the new strategy involves in situ self-assembly of multiple components during the extraction process, forming a multinuclear complex, in contrast to the mononuclear complex in most traditional systems. The advantage of such self-assembled systems lies in that the tiny differences in single metal–ligand interactions can be amplified by the multivalent cooperativity effect, which is beneficial in the separation of metal ions with similar properties. Our early work has demonstrated the successful separation of many lanthanide pairs through selective crystallization of lanthanide borates^16^. The magnification of the subtle bonding difference during crystallization is believed to be the main driving force for the high selectivity.

**Fig. 2:**
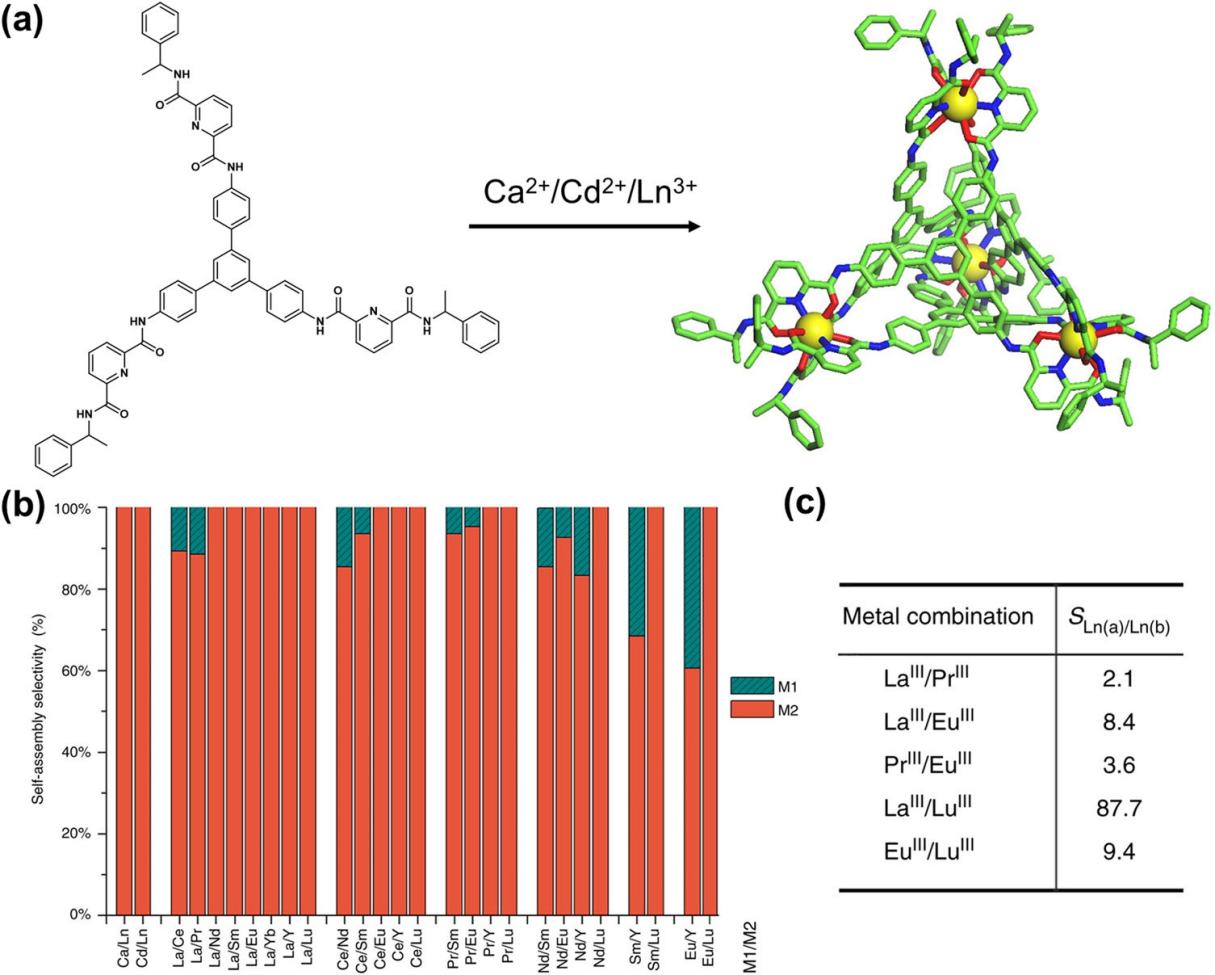
Multivalent cooperative enhancement of metal ion selectivity. **a** Molecular structure of the tris-tridentate ligand (**L**^**I**^) and its self-assembled tetrahedral cage M_4_L_4_ (M for metal ions). **b** Selective self-assembly of **L**^**I**^ with M2 over M1 in the mixture of M1/M2 determined by ^1^H NMR spectroscopy with ±5% error. **c** Separation factors (with ±5% error) from lanthanide solvent extraction experiment. Reproduced from ref. ^17^.

Sun and co-workers reported a significant cooperative enhancement of metal ion selectivity on metal–organic cages self-assembled from a tris-tridentate ligand (**L**^**I**^) with a variety of metal ions (Fig. 2a)^17^. A tetrahedral cage in which all metal centers adopt nine-coordinate tricapped trigonal prismatic geometry is formed when reacting one equivalent of ligand with metal salt. This versatile ligand shows high discrimination between metal ions with identical coordination geometries and extremely small differences in ionic radii (Fig. 2b). For example, complete binding tendency toward Eu^3+^ occurs when treating one equivalent of **L**^**I**^ with La^3+^/Eu^3+^ (1/1, molar ratio), a pair with only 0.1 Å radius difference. In general, a preference for smaller metal ions is found and the selectivity increases with the increase of ionic radii difference. The authors rationalize the results as a result of the supramolecular multivalent cooperativity, the structural stability of the cage, and favorable coordination sites. Referring to the picture in monophase reaction, the theoretical separation factor (SF) of most lanthanide pairs should reach several hundred in ideal conditions, as probed by nuclear magnetic resonance spectroscopy. However, affected by the poor water stability of the cage, the actual SF values (2.1–87.7, Fig. 2c) in extraction experiments are relatively lower than expected. Although comparable to most traditional solvent extraction systems^18^, the present results imply a big margin for improvement by employing more stable tetrahedral frameworks. Control experiments applying mono- and bis-tridentate analogs were performed to construct similar cages but resulted in poor performance in stability and selectivity, which highlights the significance of the ligand design in such an exquisite self-assembled system. This study shows how to make use of multivalent cooperativity in a self-assembled system for metal ion separation and provides new insights into the design of next-generation extractants for lanthanide separation.

**Fig. 3:**
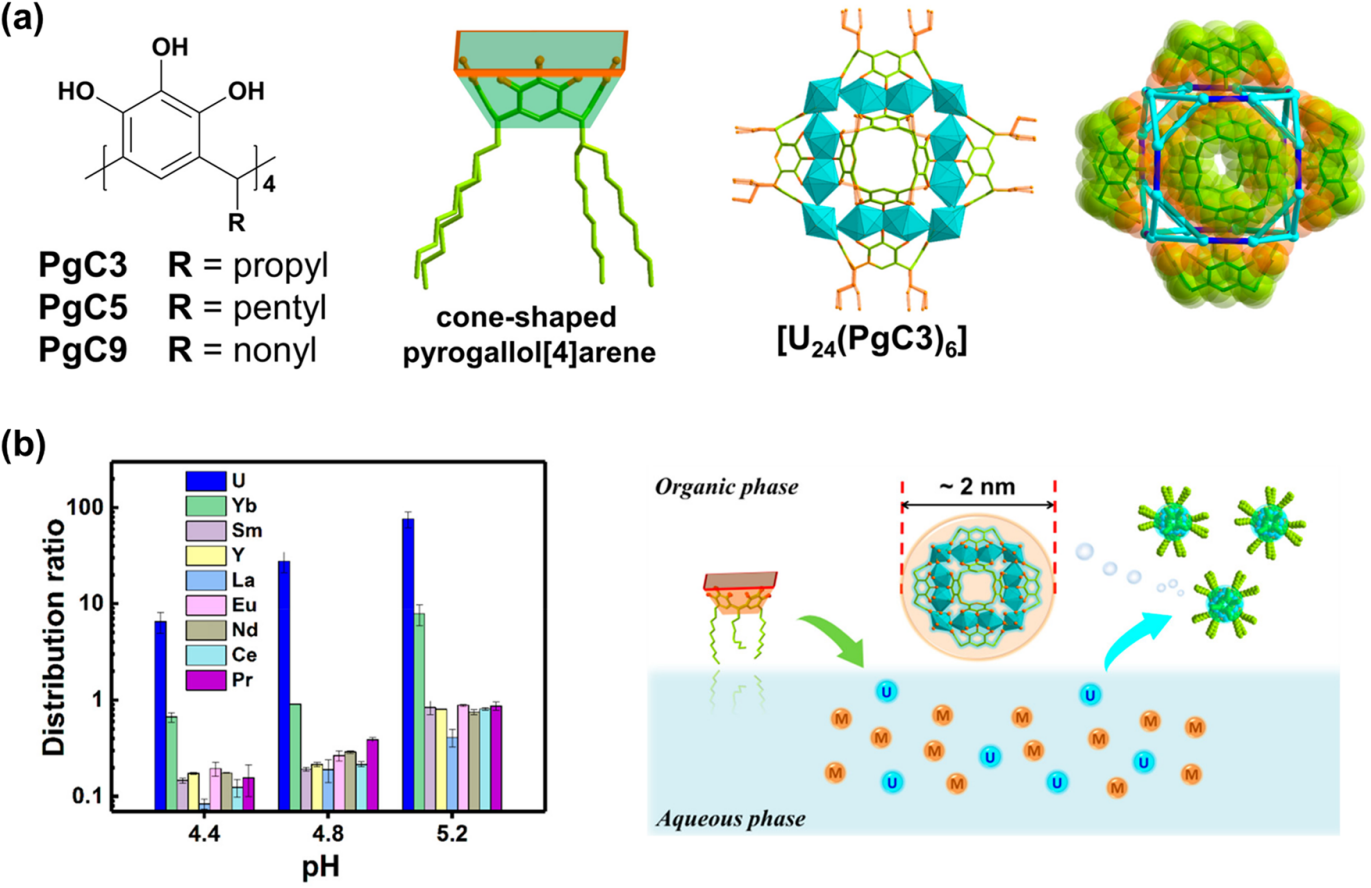
Actinide separation by self-assembled metal–organic nanocages. **a** Molecular structure of pyrogallol[4]arene (**PgC**_**n**_, *n* = 3, 5, 9) and the crystal structure of the uranyl complex [U_24_(**PgC**_**3**_)_6_]. **b** Distribution ratios (errors ranging from 0.3 to 35.7%) for uranyl and lanthanide ions at different pH and cartoon representation of selective assembly of nanocages during extraction. Reproduced with permission from ref. ^19^. Copyright 2020 American Chemical Society.

Actinide separation is crucial for recovery and recycling of key radionuclides in the used nuclear fuel. Shi, Mei, and co-workers proposed a nanoextraction strategy for efficient separation of actinides by multivalent assembly of well-defined metal–organic nanoclusters^19^. The macrocyclic ligand pyrogallol[4]arene (**PgC**_**n**_) can chelate with uranyl ions to afford a hexameric coordination cage [(UO_2_)_24_(H_2_O)_24_(**PgC**_**n**_)_6_] ([U_24_(**PgC**_**n**_)_6_], Fig. 3a) both in solution and the solid state. Generally, the exquisite multinuclear assembly imposes strict requirements on both the molecular geometries of the ligands and their coordination preference for the targeted metal ions and thus biases the desired metal ions and excludes the undesired ones. Along this path, comprehensive biphasic extraction studies were carried out. Notably, the **PgC**_**9**_ shows two orders of magnitude higher distribution coefficient toward uranyl than most of the trivalent lanthanides (Fig. 3b) under optimized conditions, and the extracted species are characterized to be identical to the hexameric cage [U_24_(**PgC**_**n**_)_6_]. This result indicates that the solvent extraction mechanism follows the in situ assembly of multi-components to precise uranyl-polyphenolic clusters, and efficient separation is achieved without the necessity for preassembly of the clusters. It is worth mentioning that such a giant entity is fabricated simply by mixing the uranyl salt with pre-designed **PgC**_**n**_ under solvothermal condition. As a comparison, traditional organic synthesis would demand much more efforts to reach this level of complexity. A representative case comes from Rebek’s highly preorganized ligand^20^ that challenges researchers by its overcomplicated stereochemistry architecture but, in effect, contains only six valid binding sites. This work offers excellent paradigm for how to construct complex framework by small units in a self-assembled way.

## Outlook

The assembly of elaborate nano-systems with a precision analogous to Nature examples has long been a topic for extensive investigation. In comparison to the bio-assemblies, the self-assembly-induced multivalent cooperativity shown here are still in the infancy stage. Nevertheless, the above cases guide the way on how to build the complex from the simple and lever the big by the small. Moreover, this idea might also be applicable to separate other metal ions or chemical feedstocks. Future efforts are expected in optimizing the ligand design for improved efficiency and creating architectures of even greater complexity.
